# Deconstructing Genetic Contributions to Autoimmunity in Mouse Models

**DOI:** 10.1371/journal.pbio.0020220

**Published:** 2004-08-17

Given the overwhelming complexity of the immune system, it's no wonder that unraveling the mechanisms responsible for immunological disease has proved so difficult. The factors that trigger autoimmunity—which involves a breakdown in the body's ability to tolerate its own molecules—are not well understood, though animal studies show that genetic predisposition greatly increases risk. And that's where the real challenge begins. For certain diseases, individuals with defects in both copies of a specific gene invariably develop the disease. But more often, diseases with an inherited component result from the complex interplay of a variety of genes, each contributing a small effect that is typically dependent both on the expression of other genes and on both random and environmental factors.

Researchers have increasingly turned to mice to model autoimmunity in humans and have found the same genetic complexity at work, with different strains of lupus-prone mice having different genes predisposing them to autoimmune disease. One model involves targeted disruption of candidate immune system genes to study their role in disease. Gene targeting experiments modify or remove a gene of interest and then watch for corresponding effects on the organism's physiology. Interpretations of results from these experiments have traditionally been predicated on the assumption that the “background,” or nontargeted, genes do not contribute to observed physiological changes. Yet in some studies, mice without targeted mutations develop an unexpected susceptibility to the autoimmune disease under study.[Fig pbio-0020220-g001]

**Figure pbio-0020220-g001:**
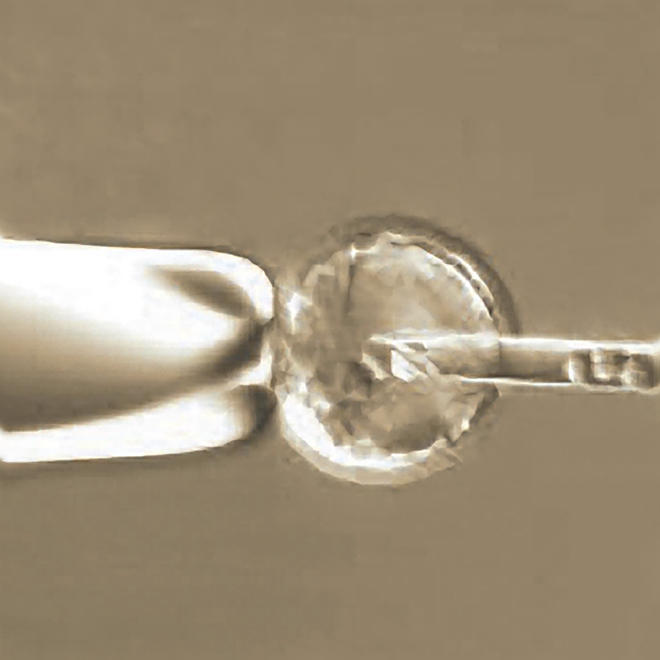
Genetic background can induce autoimmunity in knock-out mice

To investigate what effects background genes might be having in these mouse models, Marina Botto and colleagues compared the genomes of three hybrid strains of the most commonly used genetic background—the 129 and C57BL/6 hybrid mice. One of the hybrids, which carries a mutation in both copies of the *Apcs* gene, was chosen as an example of a gene-targeted model that develops a lupus-like disease, offering an opportunity to examine the relative contributions of the targeted versus background genes. *Apcs* is a candidate gene for human systemic lupus erythematosus (SLE), a form of autoimmunity marked by chronic inflammation resulting from a sustained attack on antibodies throughout the body.

Botto and colleagues found that several genomic regions from both the 129 and C57BL/6 mice contributed to autoimmunity, even in the absence of gene-targeted mutations. All of the hybrid strains developed autoimmunity, though disease was more severe in mice with *Apcs* mutations. Disease outcome in gene-targeted mice, it turns out, can be influenced not just by disease-susceptible (or disease-resistant) gene variants near the targeted gene (in this case, *Apcs*) but by the random heterogeneity of the hybrid genetic background: multiple combinations of genes in the hybrids can produce the same result.

These results fall in line with mounting evidence that background genes are not silent partners in gene-targeted disease models, but can themselves facilitate expression of the disease. This finding underscores the notion that genes are not solitary, static entities; their expression often depends on context. With genetically complex diseases, having the requisite combination of susceptibility genes does not always lead to disease. Much work remains to be done to identify the triggers that cause the immune system to turn on itself. For more on mouse models and autoimmunity, see the primer by Morel in this issue.

